# Supplementary material for: Tumor Marker B7-H6 Bound to the Coiled Coil Peptide-Polymer Conjugate Enables Targeted Therapy by Activating Human Natural Killer Cells
Source: Biomedicines. 2021 Nov 2;9(11):1597. doi: 10.3390/biomedicines9111597 (PMC8615638; doi:10.3390/biomedicines9111597)
Supplement: Supplementary file 1 [file biomedicines-09-01597-s001.zip › biomedicines-1411952-supplementary.pdf]

biomedicines

Article

Tumor Marker B7-H6 Bound to the Coiled Coil Peptide-Polymer Conjugate Enables Targeted Therapy by Activating Human Natural Killer Cells

Barbora Kalousková, Ondřej Skořepa, Denis Cmunt, Celeste Abreu, Kateřina Krejčová, Jan Bláha, Irena Sieglová, Vlastimil Král, Milan Fábry, Robert Pola, Michal Pechar, Ondřej Vaněk

Supplementary Materials

Supplementary Methods

1. Preparation of PolCC+ Polymer-peptide Conjugate

1.1 Materials

Methacryloyl chloride, 1-aminopropan-2-ol, 3-aminopropanoic acid, 4,5-dihydrothiazole-2-thiol, dimethylaminopyridine (DMAP), 1-ethyl-3-(3-dimethylaminopropyl)carbodiimide hydrochloride (EDC), N,N'-dicyclohexylcarbodiimide (DCC), 2,2'-azobis(isobutyronitrile) (AIBN), 4-cyano-4-thiobenzoylsulfanylpentanoic acid, N-(2-aminoethyl)biotinamide hydrobromide (biotin-NH2), N,N-dimethylacetamide (DMA), dimethyl sulfoxide (DMSO) and tert-butyl alcohol were purchased from Sigma-Aldrich, MT, USA. 2,2'-Azobis(4-methoxy-2,4-dimethylvaleronitrile) (V-70) was purchased from Wako Chemicals Europe GmbH, Germany. 2-Chlorotrityl chloride resin and protected amino acid derivatives were purchased from Iris Biotech, GmbH, Germany. [(1R,8S)-9-bicyclo[6.1.0]non-4-ynyl]methyl N-[2-[2-(2-aminoethoxy)ethoxy]ethyl]carbamate (Bcn-Peg2-NH2) was purchased from SiChem GmbH, Germany. 3-Amino-1-(11,12-didehydrodibenzo[b,f]azocin-5(6H)-yl)propan-1-one (Dbco-NH2) was purchased from Click Chemistry Tools, AZ, USA. All other reagents and solvents were purchased from Sigma-Aldrich, MT, USA. All chemicals and solvents were of analytical grade.

The contents of thiazolidine-2-thione (TT) groups, dithiobenzoate (DTB) end groups, and aza-dibenzocyclooctyne (Dbco) groups were determined spectrophotometrically on a Helios Alpha UV/VIS spectrophotometer (Thermospectronic, UK) using the following absorption coefficients: εTT = 10300 $L.mol^{-1}.cm^{-1}$ (305 nm, methanol), εDTB = 12100 $L.mol^{-1}.cm^{-1}$ (302 nm, methanol), $\varepsilon Dbco$ = 13000 $L.mol^{-1}.cm^{-1}$ (292 nm, methanol).

Monitoring of the conjugation reactions of Dbco-NH_2, Bcn-Peg_2-NH_2, biotin-NH_2, and peptide azide EKE to the reactive polymer precursors was performed by HPLC using a 100 × 4.6 mm Chromolith Performance RP-18e column (Merck, Germany) and a linear gradient of water/acetonitrile (0–100 % acetonitrile) in the presence of 0.1 % TFA with a UV-Vis diode array detector (Shimadzu, Japan).

The determination of the molecular weights and polydispersity of the copolymers was performed by SEC on an HPLC system (Shimadzu, Japan) equipped with UV, differential refractive index, and multi-angle light scattering (LS) DAWN Heleos II (Wyatt Technology Corp., USA) detectors using a TSK 3000 SW_{XL} column (Tosoh Bioscience, Japan) (80 % methanol, 20 % 0.3 M acetate buffer pH 6.5) at a flow rate of 0.5 ml/min.

1.2 Preparation of Monomers

N-(2-Hydroxypropyl)methacrylamide (HPMA) and 3-methacrylamidopropanoylthiazolidine-2-thione (Ma-AP-TT) were prepared as described in references [86] and [87], respectively.

1.3 Preparation of Reactive Polymer Precursor

Copolymer poly(HPMA-co-Ma-AP-TT) (P-TT) was prepared by reversible addition−fragmentation chain transfer (RAFT) copolymerization of HPMA (1.0 g, 6.98 mmol, 90 mol. %) and Ma-AP-TT (200 mg, 0.78 mmol, 10 mol. %) using V70 (9.70 μmol, 2.99 mg) as an azo initiator and (1-cyano-1-methyl-ethyl) benzenecarbodithioate (19.4 μmol, 4.29 mg)

as a chain transfer agent (CTA). The molar ratio of monomers/CTA/initiator used was 800:2:1. The polymerization mixture was dissolved in tert-butyl alcohol with 15 % DMA (11.1 ml, 0.9 M solution of monomers), transferred into a glass ampule, bubbled with Ar, and sealed. After 16 h at 40 °C, the product was isolated by precipitation with acetone, washed with diethyl ether, and dried under vacuum. The copolymer was reacted with AIBN (10 molar excess) in DMA (15 % w/w solution of polymer) under Ar for 3 h at 70 °C in a sealed ampule to remove dithiobenzoate (DTB)ω-end groups [88]. The reaction mixture was isolated by precipitation with acetone; the precipitate was washed with diethyl ether and dried under vacuum to yield copolymer P-TT (0.8 g, 67 %). Molecular parameters of the product were M_w of 95500 g.mol^{-1} and M_w/M_n of 1.06. The content of the reactive TT groups was 7.0 mol. %.

1.4 Modification of Reactive Polymer Precursor with Biotin

Copolymer P-TT (50 mg, 23 μmol TT) in DMA (0.5 ml) and N-(2-aminoethyl)biotinamide hydrobromide (1.22 mg, 3.3 μmol) in DMA (0.2 ml) were mixed and reacted for 3 h at 25 °C. Then, Bcn-Peg$_2$-NH$_2$ (2.68 mg, 8.3 μmol) was dissolved in the reaction mixture, and DIPEA (5 μl, 32 μmol) was added. The reaction was completed after 3 h (as indicated by HPLC), the remaining TT groups were removed by adding 1-aminopropan-2-ol (1.8 μl). The polymer product was twice precipitated 5 min later into acetone/diethyl ether (1:1) to yield 48 mg of the desired copolymer P-biotin-Bcn. The biotin content was 1.0 mol. %, as determined by HABA assay [89].

1.5 Attachment of Coiled Coil Peptides to the Polymer-biotin Precursor

Solution of copolymer P-biotin-Bcn (9.4 mg) in DMA (100 μl) and solution of the peptide azide (3.3 mg, 1 μmol) in DMA (50 μl) were mixed and reacted for 16 h at 25 °C. The progress of the cycloaddition was monitored by HPLC. Precipitation of the reaction mixture with acetone – ether (2:1) yielded 12.7 mg of the desired polymer-peptide conjugate. Molecular parameters of the product were M_w of 130000 g.mol^{-1} and M_w/M_n of 1.28. The content of the peptide was 26 % w/w.

Supplementary data

2. Statistical analysis of NK cell activation with B7-H6CC and B7-H6CC:PolCC

The percentage of activated NK cells was assessed by the flow cytometry of NK cells stained with antiCD107a antibody and FlowJo software data analysis. The Mann-Whitney U test with a two-tailed hypothesis was used to compare the means. The result of all mean comparisons is shown in Supplementary Figure 1.

	No activation	Plate without protein	PolCC	B7-H6 5 pmol	B7-H6 50 pmol	B7-H6CC 5 pmol	B7-H6CC 50 pmol	B7-H6CC 5 pmol + PolCC	B7-H6CC 50 pmol + PolCC
Plate without protein	** ** **								
PolCC	** ** **	n.s. ** **							
B7-H6 5 pmol	* * **	* * **	* * **						
B7-H6 50 pmol	** * **	** * **	** * **	n.s. n.s. **					
B7-H6CC 5 pmol	** ** **	** ** **	** ** **	* * *	n.s. n.s. **				
B7-H6CC 50 pmol	** ** **	** ** **	** ** **	* * **	** n.s. n.s.	** n.s. **			
B7-H6CC 5 pmol + PolCC	* ** **	* ** **	* ** **	n.s. * **	n.s. * *	n.s. ** **	* ** n.s.		
B7-H6CC 50 pmol + PolCC	* ** **	* ** **	* ** **	n.s. * **	* n.s. **	* n.s. **	n.s. n.s. **	n.s. ** **	
Chemical activation	** ** **	** ** **	** ** **	* * **	** * **	** * **	** * **	* * **	* ** **

(a)

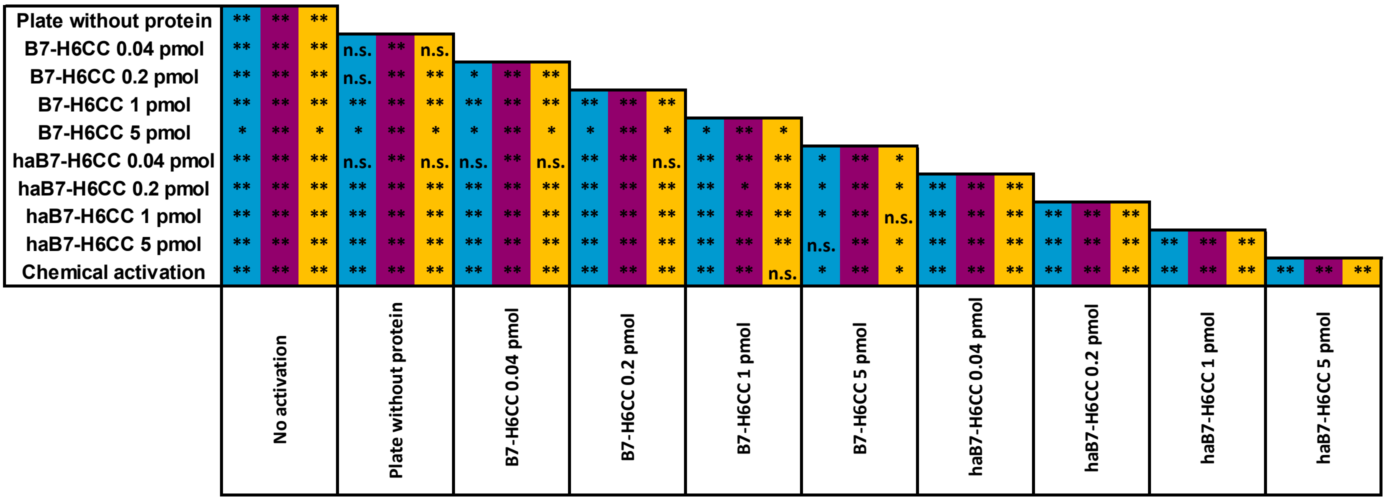

(b)

Supplementary Figure S1. Statistical analysis of NK cell activation with B7-H6 protein variants. (a) Statistical analysis of the first experiment comparing the ability of B7-H6, B7-H6CC, or B7-H6CC with PolCC to induce NK cell activation. (b) Statistical analysis of the second experiment comparing activation potential of B7-H6CC versus haB7-H6CC. n.s. stands for non-significant difference of the means, relevant p values are indicated with asterisks (** $p < 0.05$; * $p < 0.10$).

3. Global trends in NK cell activation with B7-H6CC and B7-H6CC:PolCC

To present global data analysis of NK cell activation instead of presenting results for individual donors, we constructed the graph representing a relative percentage of activated NK cells in each experiment. The baseline (activation in condition without protein) was subtracted from all conditions, and the chemical activation was considered as maximal activation (100 %). With this approach, we overcome the individual attributes of the donors, namely the different cellular sensitivity given by the magnitude between control conditions (negative control – plate without protein, positive control – chemical activation). The global trend in NK cell activation is represented in Supplementary Figure 2. The mean of technical triplicate was used in all calculations, and values from three independent donors were averaged.

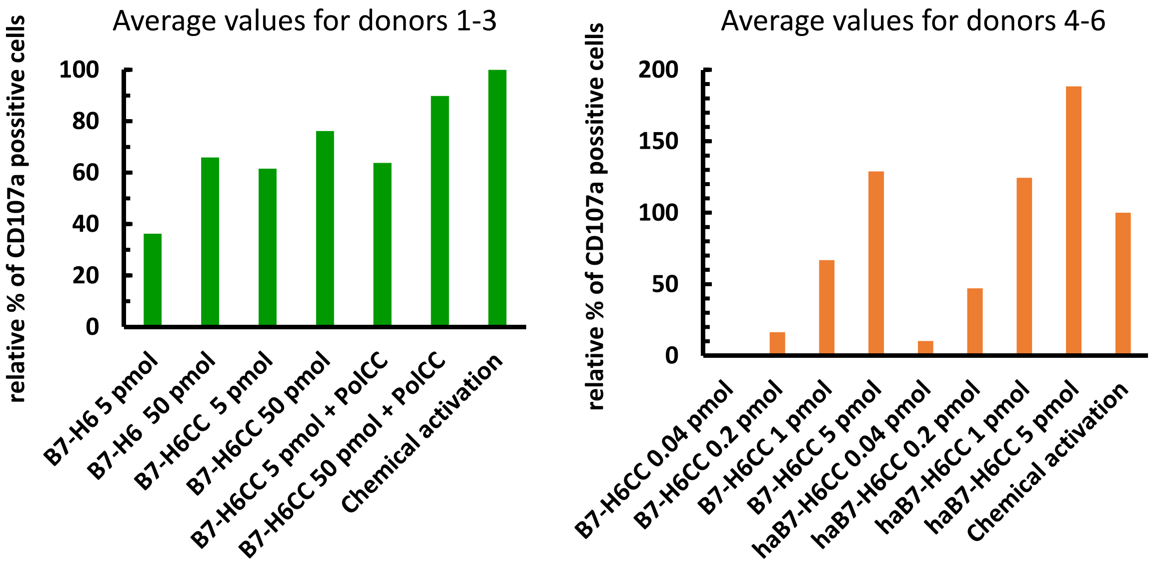

(a) **(b)**

Supplementary Figure S2. Average values of NK cell activation present the global trend. **(a)** Global analysis with the data from the donor 1-3 comparing B7-H6 activation with B7-H6CC; **(b)** analysis comparing average values of B7-H6CC and haB7-H6CC activation in the experiment with donors 4-6.

References

1. Vesely, M.D.; Kershaw, M.H.; Schreiber, R.D.; Smyth, M.J. Natural Innate and Adaptive Immunity to Cancer. *Annu. Rev. Immunol.* **2011**, *29*, 235–271, https://doi.org/10.1146/annurev-immunol-031210-101324.
2. Dunn, G.P.; Bruce, A.T.; Ikeda, H.; Old, L.J.; Schreiber, R.D. Cancer immunoediting: From immunosurveillance to tumor escape. *Nat. Immunol.* **2002**, *3*, 991–998, https://doi.org/10.1038/ni1102-991.
3. Smyth, M.J.; Dunn, G.P.; Schreiber, R.D. Cancer Immunosurveillance and Immunoediting: The Roles of Immunity in Sup-pressing Tumor Development and Shaping Tumor Immunogenicity. *Adv. Immunol.* **2006**, *90*, 1–50, https://doi.org/10.1016/s0065-2776(06)90001-7.
4. Aguilar, O.A.; Mesci, A.; Ma, J.; Chen, P.; Kirkham, C.L.; Hundrieser, J.; Voigt, S.; Allan, D.S.; Carlyle, J.R. Modulation of Clr Ligand Expression and NKR-P1 Receptor Function during Murine Cytomegalovirus Infection. *J. Innate Immun.* **2015**, *7*, 584–600, https://doi.org/10.1159/000382032.
5. Chen, D.S.; Mellman, I. Elements of cancer immunity and the cancer-immune set point. Nature **2017**, *541*, 321–330, https://doi.org/10.1038/nature21349.
6. Zitvogel, L.; Tesniere, A.; Kroemer, G. Cancer despite immunosurveillance: Immunoselection and immunosubversion. *Nat. Rev. Immunol.* **2006**, *6*, 715–727, https://doi.org/10.1038/nri1936.
7. Liu, Y.; Cao, X. Immunosuppressive cells in tumor immune escape and metastasis. *J. Mol. Med.* **2015**, *94*, 509–522, https://doi.org/10.1007/s00109-015-1376-x.
8. Dunn, G.P.; Old, L.J.; Schreiber, R.D. The Three Es of Cancer Immunoediting. *Annu. Rev. Immunol.* **2004**, *22*, 329–360, https://doi.org/10.1146/annurev.immunol.22.012703.104803.
9. Pascal, V.; Schleinitz, N.; Brunet, C.; Ravet, S.; Bonnet, E.; Lafarge, X.; Touinssi, M.; Reviron, D.; Viallard, J.F.; Moreau, J.F.; et al. Comparative analysis of NK cell subset distribution in normal and lymphoproliferative disease of granular lymphocyte conditions. *Eur. J. Immunol.* **2004**, *34*, 2930–2940, https://doi.org/10.1002/eji.200425146.
10. Bryceson, Y.T.; Björkström, N.K.; Mjösberg, J.; Ljunggren, H. Natural Killer Cells. In *The Autoimmune Diseases*; Elsevier: Amsterdam, The Netherlands, 2020; pp. 229–242.
11. Morvan, M.; Lanier, L.L. NK cells and cancer: You can teach innate cells new tricks. *Nat. Rev. Cancer* **2015**, *16*, 7–19, https://doi.org/10.1038/nrc.2015.5.
12. Shimasaki, N.; Jain, A.; Campana, D. NK cells for cancer immunotherapy. *Nat. Rev. Drug Discov.* **2020**, *19*, 200–218, https://doi.org/10.1038/s41573-019-0052-1.
13. Hu, W.; Wang, G.; Huang, D.; Sui, M.; Xu, Y. Cancer Immunotherapy Based on Natural Killer Cells: Current Progress and New Opportunities. *Front. Immunol.* **2019**, *10*, 1205, https://doi.org/10.3389/fimmu.2019.01205.
14. Habif, G.; Crinier, A.; André, P.; Vivier, E.; Narni-Mancinelli, E. Targeting natural killer cells in solid tumors. *Cell. Mol. Immunol.* **2019**, *16*, 415–422, https://doi.org/10.1038/s41423-019-0224-2.
15. Farkona, S.; Diamandis, E.P.; Blasutig, I.M. Cancer immunotherapy: The beginning of the end of cancer? *BMC Med.* **2016**, *14*, 73, https://doi.org/10.1186/s12916-016-0623-5.
16. Vivier, E.; Ugolini, S.; Blaise, D.; Chabannon, C.; Brossay, L. Targeting natural killer cells and natural killer T cells in cancer. *Nat. Rev. Immunol.* **2012**, *12*, 239–252, https://doi.org/10.1038/nri3174.
17. Chauhan, S.K.S.; Koehl, U.; Kloess, S. Harnessing NK Cell Checkpoint-Modulating Immunotherapies. *Cancers* **2020**, *12*, 1807, https://doi.org/10.3390/cancers12071807.
18. Dahlberg, C.I.M.; Sarhan, D.; Chrobok, M.; Duru, A.; Alici, E. Natural Killer Cell-Based Therapies Targeting Cancer: Possible Strategies to Gain and Sustain Anti-Tumor Activity. *Front. Immunol.* **2015**, *6*, 605, https://doi.org/10.3389/fimmu.2015.00605.
19. Pfefferle, A.; Huntington, N.D. You Have Got a Fast CAR: Chimeric Antigen Receptor NK Cells in Cancer Therapy. *Cancers* **2020**, *12*, 706, https://doi.org/10.3390/cancers12030706.
20. Liu, E.; Marin, D.; Banerjee, P.; Macapinlac, H.A.; Thompson, P.; Basar, R.; Kerbauy, L.N.; Overman, B.; Thall, P.; Kaplan, M.; et al. Use of CAR-Transduced Natural Killer Cells in CD19-Positive Lymphoid Tumors. *New Engl. J. Med.* **2020**, *382*, 545–553, https://doi.org/10.1056/nejmoa1910607.
21. Nigro, C.L.; Macagno, M.; Sangiolo, D.; Bertolaccini, L.; Aglietta, M.; Merlano, M.C. NK-mediated antibody-dependent cell-mediated cytotoxicity in solid tumors: Biological evidence and clinical perspectives. *Ann. Transl. Med.* **2019**, *7*, 105–105, https://doi.org/10.21037/atm.2019.01.42.
22. Tay, S.S.; Carol, H.; Biro, M. TriKEs and BiKEs join CARs on the cancer immunotherapy highway. *Hum. Vaccines Immunother.* **2016**, *12*, 2790–2796, https://doi.org/10.1080/21645515.2016.1198455.
23. Gleason, M.K.; Ross, J.A.; Warlick, E.D.; Lund, T.C.; Verneris, M.R.; Wiernik, A.; Spellman, S.; Haagenson, M.D.; Lenvik, A.J.; Litzow, M.R.; et al. CD16xCD33 bispecific killer cell engager (BiKE) activates NK cells against primary MDS and MDSC CD33+ targets. *Blood* **2014**, *123*, 3016–3026, https://doi.org/10.1182/blood-2013-10-533398.
24. Felices, M.; Lenvik, T.R.; Davis, Z.B.; Miller, J.S.; Vallera, D.A. Generation of BiKEs and TriKEs to Improve NK Cell-Mediated Targeting of Tumor Cells. In *Natural Killer Cells*; Humana Press, New York, NY, USA, 2016; Volume 1441, pp. 333–346, https://doi.org/10.1007/978-1-4939-3684-7_28.
25. Sarhan, D.; Brandt, L.; Felices, M.; Guldevall, K.; Lenvik, T.; Hinderlie, P.; Curtsinger, J.; Warlick, E.; Spellman, S.R.; Blazar, B.R.; et al. 161533 TriKE stimulates NK-cell function to overcome myeloid-derived suppressor cells in MDS. *Blood Adv.* **2018**, *2*, 1459–1469, https://doi.org/10.1182/bloodadvances.2017012369.

26. Märklin, M.; Hagelstein, I.; Koerner, S.P.; Rothfelder, K.; Pfluegler, M.S.; Schumacher, A.; Grosse-Hovest, L.; Jung, G.; Salih, H.R. Bispecific NKG2D-CD3 and NKG2D-CD16 fusion proteins for induction of NK and T cell reactivity against acute myeloid leukemia. *J. Immunother. Cancer* **2019**, *7*, 143, https://doi.org/10.1186/s40425-019-0606-0.
27. Han, Y.; Sun, F.; Zhang, X.; Wang, T.; Jiang, J.; Cai, J.; Gao, Q.; Hezam, K.; Liu, Y.; Xie, J.; et al. CD24 targeting bi-specific antibody that simultaneously stimulates NKG2D enhances the efficacy of cancer immunotherapy. *J. Cancer Res. Clin. Oncol.* **2019**, *145*, 1179–1190, https://doi.org/10.1007/s00432-019-02865-8.
28. Chester, C.; Fritsch, K.; Kohrt, H.E. Natural Killer Cell Immunomodulation: Targeting Activating, Inhibitory, and Co-stimulatory Receptor Signaling for Cancer Immunotherapy. *Front. Immunol.* **2015**, *6*, 601, https://doi.org/10.3389/fimmu.2015.00601.
29. Koch, J.; Steinle, A.; Watzl, C.; Mandelboim, O. Activating natural cytotoxicity receptors of natural killer cells in cancer and infection. *Trends Immunol.* **2013**, *34*, 182–191, https://doi.org/10.1016/j.it.2013.01.003.
30. Memmer, S.; Weil, S.; Beyer, S.; Zöller, T.; Peters, E.; Hartmann, J.; Steinle, A.; Koch, J. The Stalk Domain of NKp30 Contributes to Ligand Binding and Signaling of a Preassembled NKp30-CD3ζ Complex. *J. Biol. Chem.* **2016**, *291*, 25427–25438, https://doi.org/10.1074/jbc.M116.742981.
31. Brandt, C.S.; Baratin, M.; Yi, E.C.; Kennedy, J.; Gao, Z.; Fox, B.; Haldeman, B.; Ostrander, C.D.; Kaifu, T.; Chabannon, C.; et al. The B7 family member B7-H6 is a tumor cell ligand for the activating natural killer cell receptor NKp30 in humans. *J. Exp. Med.* **2009**, *206*, 1495–1503, https://doi.org/10.1084/jem.20090681.
32. Binici, J.; Koch, J. BAG-6, a jack of all trades in health and disease. *Cell. Mol. Life Sci.* **2013**, *71*, 1829–1837, https://doi.org/10.1007/s00018-013-1522-y.
33. Kruse, P.H.; Matta, J.; Ugolini, S.; Vivier, E. Natural cytotoxicity receptors and their ligands. *Immunol. Cell Biol.* **2013**, *92*, 221–229, https://doi.org/10.1038/icb.2013.98.
34. Wang, W.; Guo, H.; Geng, J.; Zheng, X.; Wei, H.; Sun, R.; Tian, Z. Tumor-released Galectin-3, a Soluble Inhibitory Ligand of Human NKp30, Plays an Important Role in Tumor Escape from NK Cell Attack. *J. Biol. Chem.* **2014**, *289*, 33311–33319, https://doi.org/10.1074/jbc.m114.603464.
35. Kaifu, T.; Escalière, B.; Gastinel, L.N.; Vivier, E.; Baratin, M. B7-H6/NKp30 interaction: A mechanism of alerting NK cells against tumors. *Cell. Mol. Life Sci.* **2011**, *68*, 3531–3539, https://doi.org/10.1007/s00018-011-0802-7.
36. Li, Y.; Wang, Q.; Mariuzza, R.A. Structure of the human activating natural cytotoxicity receptor NKp30 bound to its tumor cell ligand B7-H6. *J. Exp. Med.* **2011**, *208*, 703–714, https://doi.org/10.1084/jem.20102548.
37. Skořepa, O.; Pazicky, S.; Kalousková, B.; Bláha, J.; Abreu, C.; Ječmen, T.; Rosůlek, M.; Fish, A.; Sedivy, A.; Harlos, K.; et al. Natural Killer Cell Activation Receptor NKp30 Oligomerization Depends on Its *N*-Glycosylation. *Cancers* **2020**, *12*, 1998, https://doi.org/10.3390/cancers12071998.
38. Xu, X.; Narni-Mancinelli, E.; Cantoni, C.; Li, Y.; Guia, S.; Gauthier, L.; Chen, Q.; Moretta, A.; Vély, F.; Eisenstein, E.; et al. Structural Insights into the Inhibitory Mechanism of an Antibody against B7-H6, a Stress-Induced Cellular Ligand for the Natural Killer Cell Receptor NKp30. *J. Mol. Biol.* **2016**, *428*, 4457–4466, https://doi.org/10.1016/j.jmb.2016.09.011.
39. Arnon, T.I.; Markel, G.; Bar-Ilan, A.; Hanna, J.; Fima, E.; Benchetrit, F.; Galili, R.; Cerwenka, A.; Benharroch, D.; Sion-Vardy, N.; et al. Harnessing Soluble NK Cell Killer Receptors for the Generation of Novel Cancer Immune Therapy. *PLoS ONE* 2008, 3, e2150, https://doi.org/10.1371/journal.pone.0002150.
40. Kellner, C.; Maurer, T.; Hallack, D.; Repp, R.; Van De Winkel, J.G.J.; Parren, P.W.H.I.; Valerius, T.; Humpe, A.; Gramatzki, M.; Peipp, M.; et al. Mimicking an Induced Self Phenotype by Coating Lymphomas with the NKp30 Ligand B7-H6 Promotes NK Cell Cytotoxicity. *J. Immunol.* **2012**, *189*, 5037–5046, https://doi.org/10.4049/jimmunol.1201321.
41. Kellner, C.; Günther, A.; Humpe, A.; Repp, R.; Klausz, K.; Derer, S.; Valerius, T.; Ritgen, M.; Brüggemann, M.; Van De Winkel, J.G.; et al. Enhancing natural killer cell-mediated lysis of lymphoma cells by combining therapeutic antibodies with CD20-specific immunoligands engaging NKG2D or NKp30. *OncoImmunology* **2015**, *5*, e1058459, https://doi.org/10.1080/2162402x.2015.1058459.
42. Peipp, M.; Derer, S.; Lohse, S.; Staudinger, M.; Klausz, K.; Valerius, T.; Gramatzki, M.; Kellner, C. HER2-specific immunoligands engaging NKp30 or NKp80 trigger NK-cell-mediated lysis of tumor cells and enhance antibody-dependent cell-mediated cytotoxicity. *Oncotarget* **2015**, *6*, 32075–32088, https://doi.org/10.18632/oncotarget.5135.
43. Zhang, T.; Wu, M.-R.; Sentman, C.L. An NKp30-Based Chimeric Antigen Receptor Promotes T Cell Effector Functions and Antitumor Efficacy *In Vivo*. *J. Immunol.* **2012**, *189*, 2290–2299, https://doi.org/10.4049/jimmunol.1103495.
44. Wu, M.-R.; Zhang, T.; Demars, L.R.; Sentman, C.L. B7H6-specific chimeric antigen receptors lead to tumor elimination and host antitumor immunity. *Gene Ther.* **2015**, *22*, 675–684, https://doi.org/10.1038/gt.2015.29.
45. Hua, C.K.; Gacerez, A.T.; Sentman, C.L.; Ackerman, M.E. Development of unique cytotoxic chimeric antigen receptors based on human scFv targeting B7H6. *Protein Eng. Des. Sel.* **2017**, *30*, 713–721, https://doi.org/10.1093/protein/gzx051.
46. Vaněk, O.; Nálezková, M.; Kavan, D.; Borovičková, I.; Pompach, P.; Novák, P.; Kumar, V.; Vannucci, L.; Hudeček, J.; Hofbauerová, K.; et al. Soluble recombinant CD69 receptors optimized to have an exceptional physical and chemical stability display prolonged circulation and remain intact in the blood of mice. *FEBS J.* **2008**, *275*, 5589–5606, https://doi.org/10.1111/j.1742-4658.2008.06683.x.
47. Kolenko, P.; Skálová, T.; Vaněk, O.; Štěpánková, A.; Dušková, J.; Hašek, J.; Bezouška, K.; Dohnálek, J. The high-resolution structure of the extracellular domain of human CD69 using a novel polymer. *Acta Crystallogr. Sect. F Struct. Biol. Cryst. Commun.* **2009**, *65*, 1258–1260, https://doi.org/10.1107/S1744309109043152.

48. Deming, T.J.; Klok, H.-A.; Armes, S.P.; Becker, M.L.; Champion, J.A.; Chen, E.Y.-X.; Heilshorn, S.C.; van Hest, J.C.M.; Irvine, D.J.; Johnson, J.A.; et al. Polymers at the Interface with Biology. *Biomacromolecules* **2018**, *19*, 3151–3162, https://doi.org/10.1021/acs.biomac.8b01029.
49. Jo, S.D.; Nam, G.-H.; Kwak, G.; Yang, Y.; Kwon, I.C. Harnessing designed nanoparticles: Current strategies and future perspectives in cancer immunotherapy. *Nano Today* **2017**, *17*, 23–37, https://doi.org/10.1016/j.nantod.2017.10.008.
50. Aikins, M.E.; Xu, C.; Moon, J.J. Engineered Nanoparticles for Cancer Vaccination and Immunotherapy. *Accounts Chem. Res.* **2020**, *53*, 2094–2105, https://doi.org/10.1021/acs.accounts.0c00456.
51. Thangam, R.; Patel, K.D.; Kang, H.; Paulmurugan, R. Advances in Engineered Polymer Nanoparticle Tracking Platforms towards Cancer Immunotherapy—Current Status and Future Perspectives. *Vaccines* **2021**, *9*, 935, https://doi.org/10.3390/vaccines9080935.
52. Janisova, L.; Gruzinov, A.; Zaborova, O.V.; Velychkivska, N.; Vaněk, O.; Chytil, P.; Etrych, T.; Janoušková, O.; Zhang, X.; Blanchet, C.; et al. Molecular Mechanisms of the Interactions of N-(2-Hydroxypropyl)methacrylamide Copolymers Designed for Cancer Therapy with Blood Plasma Proteins. *Pharmaceutics* **2020**, *12*, 106, https://doi.org/10.3390/pharmaceutics12020106.
53. Apostolovic, B.; Danial, M.; Klok, H.-A. Coiled coils: Attractive protein folding motifs for the fabrication of self-assembled, responsive and bioactive materials. *Chem. Soc. Rev.* **2010**, *39*, 3541–3575, https://doi.org/10.1039/b914339b.
54. Utterström, J.; Naeimipour, S.; Selegård, R.; Aili, D. Coiled coil-based therapeutics and drug delivery systems. *Adv. Drug Deliv. Rev.* **2020**, *170*, 26–43, https://doi.org/10.1016/j.addr.2020.12.012.
55. Pola, R.; Laga, R.; Ulbrich, K.; Sieglová, I.; Král, V.; Fábry, M.; Kabešová, M.; Kovář, M.; Pechar, M. Polymer Therapeutics with a Coiled Coil Motif Targeted against Murine BCL1 Leukemia. Biomacromolecules **2013**, *14*, 881–889, https://doi.org/10.1021/bm3019592.
56. Wu, K.; Liu, J.; Johnson, R.N.; Yang, J.; Kopeček, J. Drug-Free Macromolecular Therapeutics: Induction of Apoptosis by Coiled-Coil-Mediated Cross-Linking of Antigens on the Cell Surface. *Angew. Chem. Int. Ed.* **2010**, *49*, 1451–1455, https://doi.org/10.1002/anie.200906232.
57. Wu, K.; Yang, J.; Liu, J.; Kopeček, J. Coiled-coil based drug-free macromolecular therapeutics: *In vivo* efficacy. *J. Control. Release* **2012**, *157*, 126–131, https://doi.org/10.1016/j.jconrel.2011.08.002.
58. Chu, T.-W.; Yang, J.; Zhang, R.; Sima, M.; Kopeček, J. Cell Surface Self-Assembly of Hybrid Nanoconjugates via Oligonucleotide Hybridization Induces Apoptosis. *ACS Nano* **2014**, *8*, 719–730, https://doi.org/10.1021/nn4053827.
59. Chu, T.-W.; Zhang, R.; Yang, J.; Chao, M.P.; Shami, P.J.; Kopeček, J. A Two-Step Pretargeted Nanotherapy for CD20 Crosslinking May Achieve Superior Anti-Lymphoma Efficacy to Rituximab. *Theranostics* **2015**, *5*, 834–846, https://doi.org/10.7150/thno.12040.
60. Zhang, L.; Fang, Y.; Yang, J.; Kopeček, J. Drug-free macromolecular therapeutics: Impact of structure on induction of apoptosis in Raji B cells. *J. Control. Release* **2017**, *263*, 139–150, https://doi.org/10.1016/j.jconrel.2016.12.025.
61. Zhang, L.; Fang, Y.; Li, L.; Yang, J.; Radford, D.C.; Kopeček, J. Human Serum Albumin-Based Drug-Free Macromolecular Therapeutics: Apoptosis Induction by Coiled-Coil-Mediated Cross-Linking of CD20 Antigens on Lymphoma B Cell Surface. *Macromol. Biosci.* **2018**, *18*, e1800224, https://doi.org/10.1002/mabi.201800224.
62. Gambles, M.T.; Li, J.; Wang, J.; Sborov, D.; Yang, J.; Kopeček, J. Crosslinking of CD38 Receptors Triggers Apoptosis of Malignant B Cells. *Molecules* **2021**, *26*, 4658, https://doi.org/10.3390/molecules26154658.
63. Pastorekova, S.; Gillies, R.J. The role of carbonic anhydrase IX in cancer development: Links to hypoxia, acidosis, and beyond. *Cancer Metastasis Rev.* **2019**, *38*, 65–77, https://doi.org/10.1007/s10555-019-09799-0.
64. Pechar, M.; Pola, R.; Laga, R.; Ulbrich, K.; Bednárová, L.; Maloň, P.; Sieglová, I.; Král, V.; Fábry, M.; Vaněk, O. Coiled Coil Peptides as Universal Linkers for the Attachment of Recombinant Proteins to Polymer Therapeutics. Biomacromolecules **2011**, *12*, 3645–3655, https://doi.org/10.1021/bm200897b.
65. Kissel, M.; Peschke, P.; Šubr, V.; Ulbrich, K.; Strunz, A.M.; Kühnlein, R.; Debus, J.; Friedrich, E. Detection and cellular localisation of the synthetic soluble macromolecular drug carrier pHPMA. Eur. J. Nucl. Med. Mol. Imaging **2002**, *29*, 1055–1062, https://doi.org/10.1007/s00259-002-0835-0.
66. Aricescu, A.R.; Lu, W.; Jones, E.Y. A time- and cost-efficient system for high-level protein production in mammalian cells. *Acta Crystallogr. Sect. D Biol. Crystallogr.* **2006**, *62*, 1243–1250, https://doi.org/10.1107/s0907444906029799.
67. Vaněk, O.; Celadova, P.; Skořepa, O.; Bláha, J.; Kalousková, B.; Dvorská, A.; Poláchová, E.; Pucholtová, H.; Kavan, D.; Pompach, P.; et al. Production of recombinant soluble dimeric C-type lectin-like receptors of rat natural killer cells. *Sci. Rep.* **2019**, *9*, 17836, https://doi.org/10.1038/s41598-019-52114-8.
68. Csaderova, L.; Debreova, M.; Radvak, P.; Stano, M.; Vrestiakova, M.; Kopacek, J.; Pastorekova, S.; Svastova, E. The effect of carbonic anhydrase IX on focal contacts during cell spreading and migration. *Front. Physiol.* **2013**, *4*, 271, https://doi.org/10.3389/fphys.2013.00271.
69. Bláha, J.; Pachl, P.; Novák, P.; Vaněk, O. Expression and purification of soluble and stable ectodomain of natural killer cell receptor LLT1 through high-density transfection of suspension adapted HEK293S GnTI⁻ cells. *Protein Expr. Purif.* **2015**, *109*, 7–13, https://doi.org/10.1016/j.pep.2015.01.006.
70. Durocher, Y. High-level and high-throughput recombinant protein production by transient transfection of suspension-growing human 293-EBNA1 cells. Nucleic Acids Res. 2002, 30, E9, https://doi.org/10.1093/nar/30.2.e9.
71. Pekar, L.; Klausz, K.; Busch, M.; Valldorf, B.; Kolmar, H.; Wesch, D.; Oberg, H.-H.; Krohn, S.; Boje, A.S.; Gehlert, C.L.; et al. Affinity Maturation of B7-H6 Translates into Enhanced NK Cell–Mediated Tumor Cell Lysis and Improved Proinflammatory

Cytokine Release of Bispecific Immunoligands via NKp30 Engagement. *J. Immunol.* **2020**, *206*, 225–236, https://doi.org/10.4049/jimmunol.2001004.
72. Scheuermann, T.H.; Brautigam, C.A. High-precision, automated integration of multiple isothermal titration calorimetric thermograms: New features of NITPIC. *Methods* **2014**, *76*, 87–98, https://doi.org/10.1016/j.ymeth.2014.11.024.
73. Zhao, H.; Piszczek, G.; Schuck, P. SEDPHAT—A platform for global ITC analysis and global multi-method analysis of molecular interactions. *Methods* **2015**, *76*, 137–148, https://doi.org/10.1016/j.ymeth.2014.11.012.
74. Brautigam, C.A. Calculations and Publication-Quality Illustrations for Analytical Ultracentrifugation Data. *Methods Enzym.* **2015**, *562*, 109–133, https://doi.org/10.1016/bs.mie.2015.05.001.
75. Rozbeský, D.; Kavan, D.; Chmelík, J.; Novák, P.; Vaněk, O.; Bezouška, K. High-level expression of soluble form of mouse natural killer cell receptor NKR-P1C(B6) in *Escherichia coli*. *Protein Expr. Purif.* **2011**, *77*, 178–184, https://doi.org/10.1016/j.pep.2011.01.013.
76. Schuck, P. Size-Distribution Analysis of Macromolecules by Sedimentation Velocity Ultracentrifugation and Lamm Equation Modeling. *Biophys. J.* **2000**, *78*, 1606–1619, https://doi.org/10.1016/s0006-3495(00)76713-0.
77. Skálová, T.; Kotýnková, K.; Dušková, J.; Hašek, J.; Koval', T.; Kolenko, P.; Novák, P.; Man, P.; Hanč, P.; Vaněk, O.; et al. Mouse Clr-g, a Ligand for NK Cell Activation Receptor NKR-P1F: Crystal Structure and Biophysical Properties. *J. Immunol.* **2012**, *189*, 4881–4889, https://doi.org/10.4049/jimmunol.1200880.
78. Skálová, T.; Bláha, J.; Harlos, K.; Dušková, J.; Koval', T.; Stránský, J.; Hašek, J.; Vaněk, O.; Dohnálek, J. Four crystal structures of human LLT1, a ligand of human NKR-P1, in varied glycosylation and oligomerization states. *Acta Crystallogr. Sect. D Biol. Crystallogr.* **2015**, *71*, 578–591, https://doi.org/10.1107/S1399004714027928.
79. Joyce, M.G.; Tran, P.; Zhuravleva, M.A.; Jaw, J.; Colonna, M.; Sun, P.D. Crystal structure of human natural cytotoxicity receptor NKp30 and identification of its ligand binding site. *Proc. Natl. Acad. Sci. USA* **2011**, *108*, 6223–6228, https://doi.org/10.1073/pnas.1100622108.
80. Bláha, J.; Kalousková, B.; Skořepa, O.; Pažický, S.; Novák, P.; Vaněk, O. High-level expression and purification of soluble form of human natural killer cell receptor NKR-P1 in HEK293S GnTI^{-} Cells. *Protein Expr. Purif.* **2017**, *140*, 36–43, https://doi.org/10.1016/j.pep.2017.07.016.
81. Herrmann, J.; Berberich, H.; Hartmann, J.; Beyer, S.; Davies, K.; Koch, J. Homo-oligomerization of the Activating Natural Killer Cell Receptor NKp30 Ectodomain Increases Its Binding Affinity for Cellular Ligands. *J. Biol. Chem.* **2014**, *289*, 765–777, https://doi.org/10.1074/jbc.m113.514786.
82. Pechar, M.; Pola, R.; Laga, R.; Braunova, A.; Filippov, S.K.; Bogomolova, A.; Bednarova, L.; Vanek, O.; Ulbrich, K. Coiled coil peptides and polymer-peptide conjugates: Synthesis, self-assembly, characterization and potential in drug delivery systems. Biomacromolecules **2014**, *15*, 2590–2599, https://doi.org/10.1021/bm500436p.
83. Matta, J.; Baratin, M.; Chiche, L.; Forel, J.M.; Cognet, C.; Thomas, G.; Farnarier, C.; Piperoglou, C.; Papazian, L.; Chaussabel, D.; et al. Induction of B7-H6, a ligand for the natural killer cell-activating receptor NKp30, in inflammatory conditions. *Blood* **2013**, *122*, 394–404, https://doi.org/10.1182/blood-2013-01-481705.
84. Wykoff, C.C.; Beasley, N.J.; Watson, P.; Turner, K.J.; Pastorek, J.; Sibtain, A.; Wilson, G.; Turley, H.; Talks, K.L.; Maxwell, P.; et al. Hypoxia-inducible expression of tumor-associated carbonic anhydrases. *Cancer Res.* **2000**, *60*, 7075–7083.
85. Sowa, T.; Menju, T.; Chen-Yoshikawa, T.F.; Takahashi, K.; Nishikawa, S.; Nakanishi, T.; Shikuma, K.; Motoyama, H.; Hijiya, K.; Aoyama, A.; et al. Hypoxia-inducible factor 1 promotes chemoresistance of lung cancer by inducing carbonic anhydrase IX expression. *Cancer Med.* **2016**, *6*, 288–297, https://doi.org/10.1002/cam4.991.
86. Ulbrich, K.; Šubr, V.; Strohalm, J.; Plocová, D.; Jelínková, M.; Říhová, B. Polymeric Drugs Based on Conjugates of Synthetic and Natural Macromolecules. I. Synthesis and Physico-Chemical Characterisation. *J. Control. Release* **2000**, *64*, 63–79, https://doi.org/10.1016/s0168-3659(99)00141-8.
87. Pola, R.; Král, V.; Filippov, S.K.; Kaberov, L.; Etrych, T.; Sieglová, I.; Sedláček, J.; Fábry, M.; Pechar, M. Polymer Cancerostatics Targeted by Recombinant Antibody Fragments to Gd2-Positive Tumor Cells. Biomacromolecules **2019**, *20*, 412–421, https://doi.org/10.1021/acs.biomac.8b01616.
88. Perrier, S.; Takolpuckdee, P.; Westwood, J.; Lewis, D.M. Versatile Chain Transfer Agents for Reversible Addition Fragmentation Chain Transfer (RAFT) Polymerization to Synthesize Functional Polymeric Architectures. Macromolecules **2004**, *37*, 2709−2717, https://doi.org/10.1021/ma035468b.
89. Green, N.M. A Spectrophotometric Assay for Avidin and Biotin Based on Binding of Dyes by Avidin. *Biochem. J.* **1965**, *94*, 23C–24C, https://doi.org/10.1042/bj0940023c.
90. Wood, C.W.; Woolfson, D.N. CCBuilder 2.0: Powerful and accessible coiled-coil modeling. Protein Sci. **2018**, *27*, 103–111, https://doi.org/10.1002/pro.3279.

© 2021 by the authors. Submitted for possible open access publication under the terms and conditions of the Creative Commons Attribution (CC BY) license (http://creativecommons.org/licenses/by/4.0/).
